# Necrotising soft tissue infection of the lower limb due to a perforated caecal carcinoma: a case report

**DOI:** 10.1186/1752-1947-8-80

**Published:** 2014-03-01

**Authors:** Simon J Timbrell, David Straiton, Elliot Breaks, Karim Sillah, Neil Khan

**Affiliations:** 1 Department of Surgery, Lincoln County Hospital, Greetwell Road, Lincoln LN2 5QY, UK; 2 Queens Medical Centre, University of Nottingham Medical School, Derby Road, Nottingham NG7 2UH, UK

**Keywords:** Caecal, Colorectal, Carcinoma, Retroperitoneal, Skin, Cellulitis, Necrotising, Leg, Thigh

## Abstract

**Introduction:**

We report the first case to our knowledge where an ascending colorectal tumour presented as a necrotising lower leg infection.

**Case presentation:**

We describe the unusual presentation of a previously unknown caecal carcinoma in a 69-year-old Caucasian man, which presented as a rapidly spreading limb infection due to a perforated caecal adenocarcinoma. This case presented a diagnostic dilemma and we document the investigation and management in our patient and compare this to the current published literature.

**Conclusions:**

Although rare, this case highlights how leg swelling and in particular, thigh and gluteal swelling, have the potential to be an unusual presentation of a caecal carcinoma.

## Introduction

We report a rare case of a caecal carcinoma presenting with right leg pain and swelling.

## Case presentation

A 69-year-old Caucasian man presented to a district general hospital with a two-day history of confusion, pyrexia of 38.5°C, rigors and a swollen right leg. His past medical history included hypertension and diet-controlled type II diabetes mellitus. He was pending outpatient investigation for incidental anaemia (haemoglobin (Hb) 7.8g/dl) discovered two weeks prior to admission.

On examination he had a grossly oedematous and tender right leg, from his thigh down to the inferior aspect of his calf with normal sensation and pulses. Cardio-respiratory and abdominal examinations were unremarkable and his thigh pain was exacerbated on flexion and abduction of his right hip.

Initial blood tests showed iron-deficiency anaemia (Hb 8.8g/dl, mean corpuscular volume (MCV) 64.0fL), raised inflammatory markers (C-reactive protein (CRP) 362mg/L, white cell count (WCC) 13.9 × 10^9^/L, neutrophil count 12.5 × 10^9^/L) and acute kidney injury (creatinine 136micromol/L (baseline 80micromol/L)). The working diagnosis was a deep vein thrombosis or cellulitis and he was started on intravenous co-amoxiclav and low-molecular-weight heparin.

**Figure 1:**
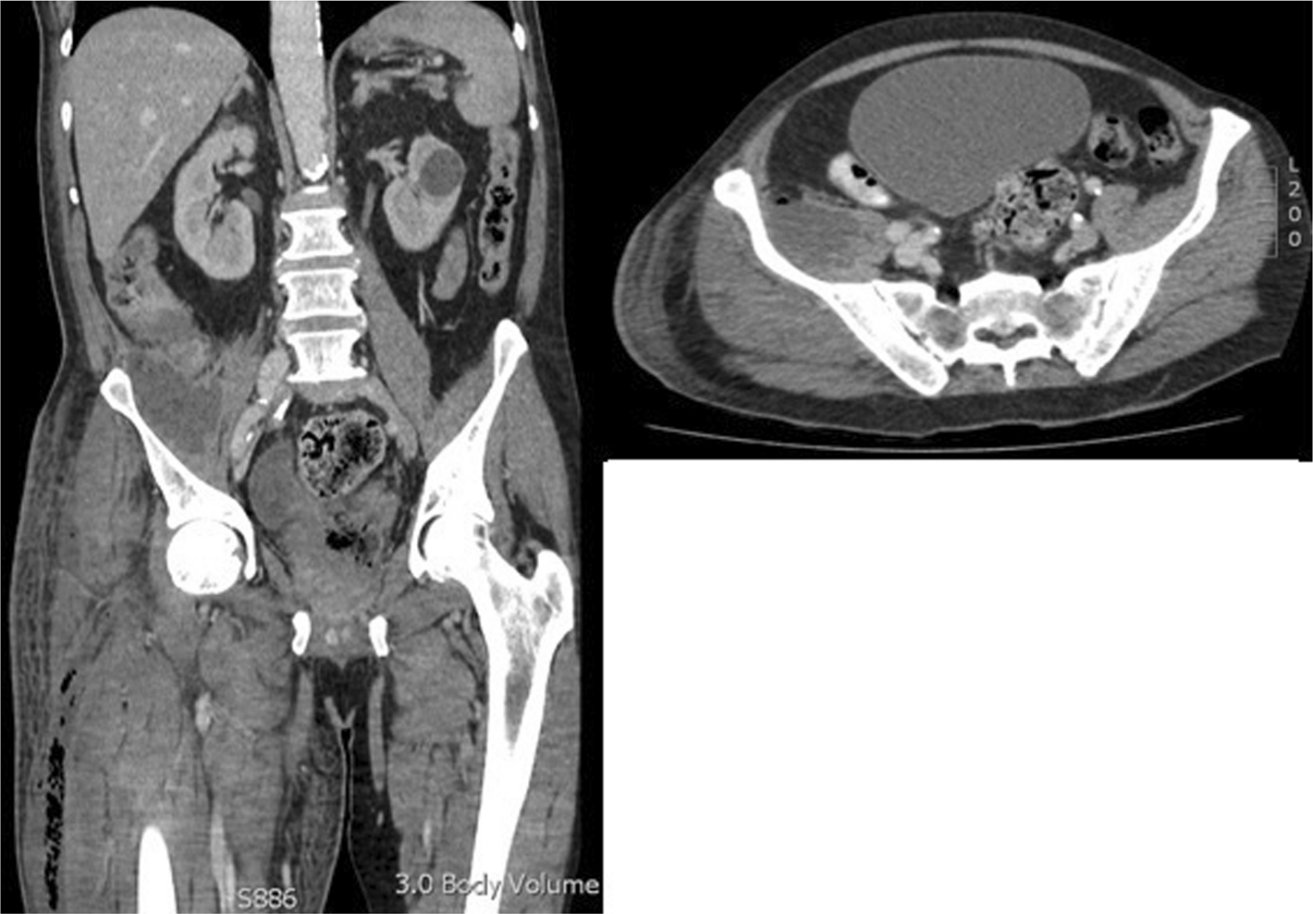
Computed tomography scan showing the perforated caecal carcinoma and free air tracking down the iliopsoas into the lower limb.

His initial investigations, including a chest X-ray, urine analysis and duplex scan, were unremarkable. A subsequent computed tomography (CT) scan of his abdomen, pelvis and both limbs showed a circumferential caecal thickening with a retroperitoneal perforation leading to a psoas abscess that involved the iliacus muscle, right groin and extended below the knee as shown in Figure 1. The whole of his right thigh was enlarged and oedematous with no bony involvement seen. There was no evidence of metastasis or free intraperitoneal fluid. A differential diagnosis of an appendicular abscess, Crohn’s disease, lymphoma or caecal carcinoma was suggested by the radiologist.

After the CT scan, a surgical opinion was sought by the admitting medical team who recommended a blood transfusion and CT-guided percutaneous drainage that obtained pus from the retroperitoneal collection. Due to his deteriorating clinical condition, our patient underwent an extensive right lateral thigh and lower leg fasciotomy and debridement down to the ankle.

During this debridement, bullae on the lateral aspect of the thigh were found and foul-smelling fluid drained, which the operating surgeons believed was consistent with necrotising fasciitis. The vastus subfascia was incised, debridement of necrotic skin was undertaken and multiple loculations of subfacial pus drained along with the insertion of a Penrose drain.

**Figure 2:**
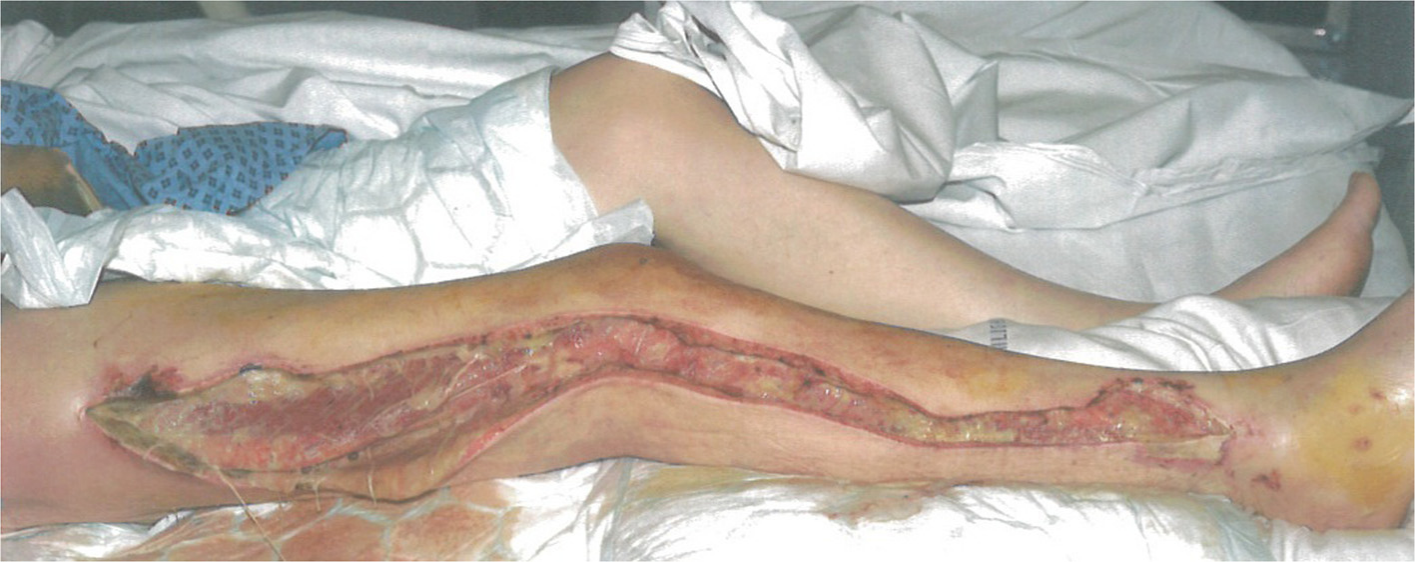
This picture demonstrates the extensive debridement necessary to remove infection from the tracking abscess.

He went to the intensive care unit (ICU) for supportive care post-operatively. Over the following six days he underwent two further lower limb debridements, where pus was found to be tracking between the ankle and thigh wounds so the incision was extended further as shown in Figure 2.

Once stabilised after a nine-day admission to the ICU, he underwent a laparoscopic defunctioning loop ileostomy to divert the enteric stream. During the five-week admission he spent nearly two weeks on the ICU and had a prolonged course of intravenous gentamicin, meropenem and clindamycin. On day 21 he had an enteroscopy via his ileostomy, where ileocaecal biopsies taken demonstrated a moderately differentiated caecal adeoncarcinoma.

His case was discussed by the colorectal multi-disciplinary team (MDT) and the decision was made to involve the tissue viability team to manage his leg wound with negative pressure dressings, allowing this to heal via secondary intention and delay any immediate abdominal surgery.

Three weeks after he was discharged from his initial admission he returned for a right hemicolectomy and defunctioning ileostomy, rather than a primary anastomosis given his poor nutritional state and recurrent lower limb wound infections. The histology confirmed a moderately differentiated caecal adenocarcinoma with a posterior perforation. None of the lymph nodes received at the time of surgery contained metastases and the tumour was therefore a Dukes B or T3, N0, M0 tumour. Our patient is currently undergoing a course of post-operative chemotherapy, given the incomplete excision, and has no signs of disease spread or recurrence.

## Discussion

Colon cancer is the third commonest type of cancer in the UK [1]. Right-sided tumours account for roughly 20 percent of colorectal carcinomas and present differently from left-sided tumours. Patients usually present with unexplained anaemia, weight loss, a right lower quadrant mass with or without perforation and appendicitis [1].

We conducted a literature review using PubMed and Google Scholar and the terms ‘colorectal’, ‘carcinoma’, ‘unusual’, ‘presentation’, ‘thigh’ and ‘leg’. This showed that buttock and thigh signs secondary to colorectal pathology usually occur in the descending colon secondary to a diverticular perforation [2]. We only found two cases where an ascending colon carcinoma presented as a right thigh abscess [3,4] and several further cases where it presented as a right gluteal abscess or cellulitis. Our literature review highlighted that a retroperitoneal abscess was more commonly associated with infection and inflammation of the duodenum, pancreas, terminal ileum, appendix, and descending colon.

The two previous single patient case reports where an ascending colon tumour presented with leg signs demonstrate how unusual this presentation is. Bohrer and Bodine documented a caecal carcinoma presenting as thigh emphysema as a result of a perforation and tracking of air through the sciatic foramen into the subcutaneous tissues in the inferior aspect of the gluteal region [3]. Mann *et al*. describe a brief three-day history of right thigh swelling in a patient secondary to a large retroperitoneal abscess that tracked along the iliopsoas into the anterior compartment of the right thigh [4].

As well as being a rare presentation the extent of the necrotising infection was far greater in the case we reported than any previously recorded. This may explain why other differentials such as a deep vein thrombosis were initially considered.

Fotiadis *et al*. described a sealed retroperitoneal perforation and abscess that presented even more insidiously with unexplained pyrexia without cutaneous signs as it did not track into the thigh [5].

We believe that our case and those outlined above highlight how a retroperitoneal perforation is an important differential to consider when patients present with other symptoms suggestive of colonic carcinoma and signs can be varied, subtle and easily missed. The importance of this is further evidenced in a case report by Kobayshi *et al*. where a 72-year-old woman with anaemia presented with right groin and buttock pain six months prior to her eventual diagnosis with colorectal carcinoma. In this case the initial infection was successfully treated conservatively with antibiotics and further investigation was only prompted six months later when the woman represented with a thigh abscess and air was obtained during drainage of this collection. This case highlights how it is easy to miss the underlying diagnosis; however, if this had been considered in the context of her anaemia, she may have undergone further investigation that would have identified the underlying carcinoma earlier [6].

Our reading highlighted physical signs that could help identify a tracking abscess secondary to a caecal perforation. These included a pyrexia of unexplained origin, psoas rigidity, a palpable mass and costolumbar sensitivity [4,6].

In terms of initial investigations, all the patients identified during our literature review presented with raised inflammatory markers and had CT scans of the abdomen and pelvis that confirmed the presence of caecal thickening, a retroperitoneal abscess +/− tracking along the illiapsoas. Kobayshi and Mann describe how an initial hip X-ray may be useful to demonstrate free air [4,6].

The majority of patients were managed as in this case with CT-guided drainage of the retroperitoneal abscess with broad spectrum antibiotic coverage. Our review also indicated that if a collection is secondary to a perforated caecal carcinoma, then a right hemicolectomy is indicated. This may be an emergency operation as reported by Mann [4] and Fotiadis [5] or a semi-elective operation as was the case here and with Kobayshi *et al.*[6]. This will obviously be influenced by the patient’s clinical condition at the time; in this scenario our patient was deemed to be too unwell to undergo a primary resection.

## Conclusions

This case highlights how a sealed, perforated caecal mass can present in unusual ways and the diagnosis should be considered in patients with extensive limb cellulitis or necrotising fasciitis and features suggestive of a caecal carcinoma. We outline a number of physical signs that may be useful in considering this as a differential diagnosis and prompt further investigation, usually in the form of a CT scan and highlight how it is an important differential not to miss, given its potential to lead to life-threatening sepsis that is unlikely to improve without intervention.

## Consent

Written informed consent was obtained from the patient for publication of this case report and any accompanying images. A copy of the written consent is available for review by the Editor-in-Chief of this journal.

## Abbreviations

CRP: C-reactive protein; CT: computed tomography; Hb: haemoglobin; ICU: intensive care unit; MCV: mean corpuscular volume; WCC: white cell count.

## Competing interests

The authors declare that they have no competing interests.

## Authors’ contributions

ST collated data and wrote the manuscript. DS collected data and reviewed the manuscript. EB contributed to the literature review and the writing of the manuscript. KS was also involved in the patient’s care and the writing of the manuscript. NK was the consultant for our patient and reviewed the manuscript. All authors read and approved the final manuscript.
